# Approaches towards optimizing individualized, high‐quality, evidence‐informed care

**DOI:** 10.1111/hex.12607

**Published:** 2017-07-17

**Authors:** Mary Chambers

**Affiliations:** ^1^ Faculty of Health Social Care and Education Kingston University and St. George's University of London

Health‐care professionals and service providers are constantly striving to find the best ways of delivering high‐quality, individualized, evidence‐informed care. Critical to the process is shared decision‐making, exchange of information and patient and public engagement at all levels from the clinical encounter to policy development. Other essential characteristics are communication, partnership working, therapeutic relationships, respect for dignity, cultural sensitivity and team working.

Manuscripts in this edition draw attention to patient information and the importance of co‐design, experiences of immigrant woman, parental involvement, the sexual needs of care home residents, discharge protocols and the contribution of technology.

There is consensus that information leaflets are helpful for patients. Information is available through other sources such as the Internet, which some patients cautiously access Zizzo et al., but others prefer written information. Xie et al., illustrate that with respect to the sources of health information used by Chinese patients with cancer and their family caregivers. The systematic review reported by Sustersic et al. describes the impact of patient information leaflets (PILs) on patients' knowledge, satisfaction and shared decision‐making. It raises a note of caution that PILs have the potential (depending on context) to increase anxiety and impact on a patient's sense of self‐efficacy. However, what appears to be missing from the review is any consideration of the importance of co‐design,^1^, ^2^ now considered essential in the production of high‐quality patient information. Engaging patients in the design and production of information materials helps ensure that the material is relevant and presented in an accessible format to facilitate uptake and usage.

Co‐design is reinforced in work of Learmonth et al. when exploring the needs of those suffering from multiple sclerosis and the low uptake of exercise. These authors report that patients were dissatisfied with the level of exercise promotion stating that “effort is required to develop a proactive partnership and structured exercise communication between patients with MS and health‐care providers.” This statement suggests a realization of the importance of co‐design without it being explicit.

When addressing delicate care issues within specific populations where relevance and cultural sensitivity are paramount, co‐design is a key. The studies of Partin et al. and Quaife et al. draw attention to this with respect to screening. In relation to patient perceptions about prostate cancer screening benefits, harms and recommendations, Partin et al. compared perceptions across race, age and other variables to inform educational materials and conclude that information targeted by race, age or prostate‐specific antigen level may not be necessary, but information addressing the misperceptions about benefits and lack of awareness of potential harms is required.

Misconceptions and mixed views were also the findings from the work of Quaife et al. when exploring attitudes towards lung cancer screening in socio‐economically deprived and heavy smoking communities. The findings from the studies of Partin et al. and Quaife et al. suggest the need for a co‐design approach when developing future educational materials to ensure relevance and usability. Reporting a study on colorectal cancer screening, Barnett et al. highlight the need for patient engagement and co‐design of any information materials to ensure that patients understand the intended message; how a negative faecal occult blood test may influence symptom recognition and future help‐seeking behaviour is also reported.

Successful patient participation in interprofessional team meetings is the focus of the paper by Jacques van Dongen et al. especially when treating those with complex health‐care needs. The authors state that patient participation during team meetings was appreciated by both patients and professionals and offer some suggestions as to how such meetings could be conducted. The need for a tailored approach is stressed, with attention paid to patients' willingness and ability to participate. The importance of team working is also highlighted in the paper by Meaney et al. when considering the impact of stillbirth on parents. Using interpretative phenomenological analysis (IPA), the authors report that in the days following stillbirth, while both parents expressed fear, mothers were driven to plan a future pregnancy while fathers were reluctant to consider any pregnancies. The authors suggest that to meet the complex needs of the mother and father, a multidimensional, multiprofessional team approach is required to include both health‐ and social care professionals during future care.

Three papers focus on older people. Zizzo et al., in their study looking at the participation preferences of patients with Parkinson's disease, note the complexities of decision‐making processes with some patients expressing a preference to take a final decision; others wanted it to be shared (patient/professional) and others that the doctor takes the decision but that they consent to it. Acknowledging self‐management as a contributing factor to independence, Veldman et al. designed a tool to evaluate the self‐management behaviour of older people within a Dutch context—Partners in Health scale for older adults. Using the original Partners in Health scale (PIA), the authors, applying psychometric principles, adapted and modified it to the context of community‐living older adults. To set the level of self‐management of older adults, reliable and valid measurement instruments are important to assess the status and to follow the development of someone's self‐management behaviour over time. This insight gives professionals and older adults the opportunity to individually tailor the level of care and support needed, help communication about need leading to better provision of efficient and tailored health care.

Syme et al., address an important and often neglected aspect of the life of older people, especially those in nursing home care. The authors state that contrary to societal beliefs, a range of sexual and intimate behaviours are exhibited by older adults in nursing home settings. Those with dementia‐related disease have the right and need to engage in healthy, intimate or sexual relationships but often not actualized in nursing home settings. This is partly due to resident's loss of autonomy, issues with the built environment, staff and family attitudes towards resident sexual expression and lack of sexual consent capacity. Consequently, nursing homes often err on the side of safety, and prohibit such activity, with family and staff assuming the right to make such decisions. Little is known about the public perspectives on sexual expression and dementia, and its impact on the development of a resident‐ or consumer‐centred policy, therefore a need for well‐informed person‐centred policy developed in partnership with all key stakeholders. The community/citizens juries and the checklist outlined by Thomas et al. could be an approach to the development of such policy with the potential to capture the views of the wider community on sexual expression in nursing homes, as well as the views of care home staff, residents and families. This inclusive approach would contribute to a safe, respectful and person‐centred environment and enable sexual expression for those living in nursing homes.

Communication is constantly referred to as one of the pillars of quality care. Ingram et al., present a positive and innovative method of encouraging communication when preparing families for the discharge of preterm infants. The “Train‐to‐Home” initiative aimed to improve parents' preparedness and confidence prior to infant discharge. Similarly, discharge communication is also a vital aspect of high‐quality emergency care as indicated by Curran et al., who, in their pilot study, developed a discharge communication coding scheme and coding manual that could be used to accurately and reliably code discharge communication between health‐care professionals and parents in a paediatric ED.

Increasingly, technology is playing a greater role in the delivery of health care offering opportunity for the development of novel and innovative, effective and efficient treatment interventions and support mechanisms. Sin et al. developed an Internet‐based approach to provide a first‐episode psychosis (FEP) sibling‐specific intervention. This intervention offers information about psychosis, as well as strategies to enhance coping, and opportunities for peer support. The qualitative study reported focuses on what factors influence successful recruitment of siblings to individuals with FEP to e‐health interventions.

Staying with mental health, the study reported by Simmons et al. considers the evaluation of a decision aid for youth depression, something, which is on the increase. It is acknowledged that this group of young people are hard to engage leading Simmons et al. to develop and evaluate a “proof of concept” online decision aid for young people experiencing mild, mild‐to‐moderate or moderate‐to‐severe depression. The conclusion was that connecting young people with effective treatment in a timely manner can help to ease the burden of depressive symptoms and minimize the potential negative aspects of depression.

A further and quite different use of technology was a new graphical format (simple proportional bar graph including stick figure icons) to communicate treatment effects to patients as described by Kasper et al. The purpose of this investigation was to evaluate the new presentation format's efficacy with regard to communicating study effects using a web‐based four‐arm randomized controlled trial. Findings suggest that the new format is advantageous may facilitate the comparison of different treatment options in comprehensive patient information. However, as the trial was exploratory, further research is needed. This is also the message from Simmons et al. and reflects the dynamic and evolving nature of health‐care technology.

This edition of Health Expectations, therefore, considers a range of approaches and methodologies reporting the quest for high‐quality, person‐centred, evidence‐informed care.
